# Exosomal lnc-CDHR derived from human umbilical cord mesenchymal stem cells attenuates peritoneal epithelial-mesenchymal transition through AKT/FOXO pathway

**DOI:** 10.18632/aging.204883

**Published:** 2023-07-18

**Authors:** Taiwei Jiao, Yuling Huang, Haiyan Sun, Lina Yang

**Affiliations:** 1 Department of Gastroenterology and Endoscopy, The First Hospital of China Medical University, Shenyang 110001, Liaoning, P.R. China; 2 Department of Geriatrics, The First Hospital of China Medical University, Shenyang 110001, Liaoning, P.R. China; 3 Department of Endodontics, School of Stomatology, China Medical University, Shenyang 110001, Liaoning, P.R. China; 4 Department of International Physical Examination Center, The First Hospital of China Medical University, Shenyang 110001, Liaoning, P.R. China

**Keywords:** peritoneal dialysis, peritoneal fibrosis, EMT, mesenchymal stem cell, exosome

## Abstract

Objective: Chronic stimulation of peritoneal dialysis (PD) fluid leads to the epithelial-mesenchymal transformation (EMT) of mesothelial cells, peritoneal fibrosis (PF), and ultimately ultrafiltration failure. Some studies have proposed that mesenchymal stem cells (MSCs) can alleviate PF. This study aimed to investigate whether the exosomes from human umbilical cord MSCs (hUMSCs) could alleviate peritoneal EMT.

Methods: Human peritoneal mesothelial cell line (HMrSV5) were treated with high glucose (HG) for 48 hours to induce the peritoneal EMT model. An inverted fluorescence microscope was used to observe the internalization of exosomes derived from hUMSCs (hUMSC-Exos). Western blot and real-time PCR were used to evaluate the expression of α-SMA, Vimentin, E-cadherin, PTEN, and AKT/FOXO3a. The relationships of lncRNA CDHR and miR-3149, miR-3149 and PTEN were detected by dual luciferase reporter gene assay.

Results: Compared with HG-induced HMrSV5, E-cadherin and PTEN levels significantly increased whereas α-SMA and Vimentin levels significantly decreased after treatment of hUMSC-CM and hUMSC-Exos (*P* < 0.05). An inverted fluorescence microscope showed HMrSV5 can absorb exosomes to alleviate EMT. Furthermore, exosomes extracted from lnc-CDHR siRNA-transfected hUMSCs can’t ameliorate HMrSV5 EMT. Moreover, both CDHR overexpressed and miR-3149 inhibitor in HG-induced HMrSV5 alleviated the expression of α-SMA, and Vimentin, and increased the expression of E-cadherin and PTEN, and AKT/FOXO3a. A rescue experiment showed that CDHR overexpressed expression was repressed by miR-3149 in the HG-induced peritoneal EMT model.

Conclusions: Exosomal lnc-CDHR derived from hUMSCs may competitively bind to miR-3149 to regulate suppression on target PTEN genes and alleviate EMT of HMrSV5 through AKT/FOXO pathway.

## INTRODUCTION

Peritoneal dialysis is one of the alternative treatments for patients with end-stage renal disease, with more than 272,000 patients receiving it worldwide, accounting for approximately 11% of the global dialysis population [1]. Chronic stimulation by dialysis fluid leads to recurrent damage and inflammatory responses in peritoneal tissue [2], oxidative stress, and apoptosis [3], which eventually leads to epithelial-to-mesenchymal transition (EMT) of mesothelial cells, causing fibrosis in peritoneal tissue and ultimately leading to ultrafiltration failure [4]. Therefore, the study of the mechanism of peritoneal EMT and its effective preventive interventions has significant theoretical and clinical implications for improving the survival quality of patients with end-stage renal disease.

Mesenchymal stem cells (MSCs) are derived from bone marrow, adipose, umbilical cord (hUMSCs), placenta, peripheral blood, etc. [5]. The therapeutic effects of MSCs mainly rely on their paracrine effects [6]. MSCs can secrete a variety of soluble trophic factors and extracellular vesicles, including microvesicles (30-150 nm), exosomes (200-1000 nm), and apoptotic vesicles (800-5000 nm). Among them, RNAs, DNAs, proteins, and lipids are transported by exosomes to deliver information to target cells.

There are numerous studies showing that MSCs can alleviate fibrotic diseases, such as pulmonary fibrosis, liver fibrosis, renal fibrosis, and cardiac fibrosis [7]. The role of MSCs in peritoneal fibrosis (PF) is also recognized. However, direct infusion of MSCs is gradually being replaced by MSC supernatant due to their potential immunogenicity and tumorigenicity [8]. Ueno T et al. demonstrated that MSCs suppressed inflammation in experimental PF through paracrine secretion [9]. Yang CY et al. further confirmed that MSCs secrete IL-6 to polarize macrophages to an M2 phenotype that attenuates dialysis-induced PF [10]. Recently, Nagasaki et al. suggested that serum-free culture conditions could enhance the antifibrotic capacity of BMMSCs in experimental PF by inhibiting inflammatory effects [11]. Compared to MSCs, exosomes overcome the disadvantages of MSCs mentioned before. Exosomes with small sizes assist in them arriving at the target easily, such as crossing the blood-brain barrier, as well as lesser immunogenicity and tumorigenicity [5].

As information molecules, ncRNAs play an important role in the development of fibrotic diseases, especially miRNA and lncRNA. miR-15a-5p/-320c/-30a all inhibit peritoneal EMT and PF [12–14]. MiR-15a-5p can inhibit human peritoneal mesothelial cell fibrosis; miR-302c overexpression attenuated PF; miR-30a overexpression blocked Snai1 and EMT and inhibited PF. LncRNA GAS5 [15] and AK089579 [16] inhibit EMT transition of peritoneal mesothelial cells by competitively binding to miR-21 and miR-296-3p respectively.

In addition, EMT is an indispensable step in fibrosis [17]. Recent findings have revealed that the FOXO signaling pathway plays an important role in organ fibrosis and EMT processes [18]. FOXO3a is a representative member of the FOXO sub-family, and an increasing number of investigations have shown that FOXO3a is closely associated with organ fibrosis and EMT processes in the heart [19]. Similarly, previous studies have indicated that the AKT signaling pathway plays an important role in the process of peritoneal EMT [20], and it has been found that AKT can regulate EMT by decreasing FOXO3a levels [21], suggesting that the AKT/FOXO signaling pathway may play an important role in the process of peritoneal EMT.

To summarize the above, we propose that exosomal lnc-CDHR derived from hUMSCs sponging miR-3149 to target PTEN alleviate peritoneal EMT via the AKT/FOXO pathway.

## MATERIALS AND METHODS

### Cell culture

HMrSV5 was purchased from GuangZhou Jennio Biotech Co., Ltd. (China). According to the instruction of cell culture of HMrSV5, HMrSV5 were cultured in MEM medium containing 10% FBS (Gibco, China) at 37° C in 5% CO2. MEM complete medium was changed every 2-3 days and then digested with 0.25% trypsin (Gibco, China) at a ratio of 1:2~ 1:3 for passaging. When the cell density reached 60-70% confluence, HMrSV5 was given with different stimulations (Control, 2.5% HG group, HG+MSC-CM group, HG+HUMSC-Exo group).

HUMSCs and stem cell culture medium (basal medium containing serum substitutes) were purchased from Shenyang Engineering Technology R&D Center of Cell Therapy Co., Ltd (Liaoning Province, China). According to three standards of MSCs [22], the plastic adhesion of hUMSCs was observed under light microscopy. MSC specific surface markers, CD105, CD34, and HLA-DR (eBioscience, USA), were identified by flow cytometry. Osteogenesis, adipogenesis, and chondrogenesis were confirmed by Alizarin Red, Oil Red, and Alcian Blue staining (Cyagen, China).

### Flow cytometry

HUMSCs were cultured in stem cell culture medium, at 37° C with an atmosphere of 5% CO2. A total of 5x10^6^ cells were prepared from each sample. hUMSCs were incubated with 5μl antibody above mentioned in 100μl PBS at 37° C protected from light. PE and FITC monoclonal mouse IgG1 K Isotype were used as negative control. HUMSC surface marker expression was evaluated by the BD LSRFortessa cell analyzer, and the acquired data were further analyzed with FACSDiva (Version 6.2).

### Exosome purification and characterization

After hUMSCs reach about 80% confluence, the supernatant was collected. Exosomes were extracted after binding, washing, and elution with differential centrifugation, according to the protocol of exosome extraction kit (Rengen Biosciences, EXOCon40-10). Exosomes were validated according to previous studies [23]. 50-100μl 2% paraformaldehyde solution was used to dissolve exosomes, and photos were taken with an electron microscope. The quantity and size of exocrine granules (Zeta View PMX110) and the movement of particles (NTA software, ZetaView 8.02.28) were measured. Exosomal markers HSP70, and TSG101 (Abcam) were determined using western blotting.

### Exosome labeling

HMrSV5 with 40% confluence are cultured with exosomes derived from hUMSCs labeled by PKH67. After 6 hours of culture, the cells were washed 3 times with PBS, and then fixed with 4% PFA for 10 minutes at room temperature. The cells were washed with PBS again and incubated with DAPI for 5 min under protection from light. Finally, the uptake of exosomes by HMrSV5 was observed under an inverted fluorescence microscope using a 40× objective lens.

### Quantitative real-time PCR (qRT-PCR)

With the aid of a reverse transcription kit (Takara, RR047A and Sangon, 532453), complementary DNA (cDNA) of mRNA and miRNA was synthesized after RNA extraction with TRIzol, following respective instructions. The primer sequences of real-time PCR (Table 1) were designed and synthesized by Sangon Biotech Co., Ltd. Mrna, and miRNA levels were quantitatively detected by the SYBR Green kit (Takara, RR820A). GAPDH was used as an internal reference for mRNA and lncRNA expression, whereas U6 was used for miRNA expression. The relative expression of the transcript was quantified with 2^-ΔΔCt^ × 100%.

**Table 1:** The primer sequences of real-time PCR.

**Target**	**Primer sequences**
GAPDH (hsa)	F: 5-gcaccgtcaaggctgagaac-3
	R: 5-tggtgaacacgccagtgga-3
E-cadherin (hsa)	F: 5-gtcactgacaccaacgataatcct-3
	R: 5-tttcagtgtggtgattacgacgtta-3
α-SMA (hsa)	F: 5-cctcccttgagaagagttacga-3
	R: 5-gatgctgttgtaggtggtttca-3
Vimentin (hsa)	F: 5-cagtcactcacctgcgaagt-3
	R: 5-agttagcagcttcaagggca-3
Lnc-CDHR-3-7 (hsa)	F: 5-AAGGGAGTACCATGCAGCTGT-3
	R: 5-GCAATGTATTCTGGCACTCTCTCC-3
U6 (hsa)	F: 5-ctcgcttcggcagcaca-3
	R: 5-aacgcttcacgaatttgcgt-3
miR-3149 (hsa)	F: 5-GCGCGTTTGTATGGATATGTGT-3
	R: 5-AGTGCAGGGTCCGAGGTATT-3

### Western blot

Collected HMrSV5 were lysed with RIPA and PMSF (100:1). The extracted protein was quantified with a BCA assay kit, and denatured with loading buffer and PBS metal bath at 100° C for 5min. 30μg protein samples were subjected to 10% or 12% SDS-PAGE, and then electro-transferred onto the FVDF membrane for 120 min at a constant current of 200 mA. Subsequently, the membrane was blocked with Protein Free Rapid Blocking Buffer (Servicebio, G2052-500) and incubated overnight from following primary antibodies: Abcam rabbit anti-α-SMA, rabbit anti-E-cadherin, rabbit anti-Vimentin, rabbit anti-PTEN, rabbit anti-FOXO3a, rabbit anti-AKT, mouse phospho-AKT, rabbit anti-Hsp70, rabbit anti-TSG101, and mouse anti-β-actin. PVDF membranes were re-probed with secondary antibodies for 1 to 2 h at room temperature and visualized by ECL (MicroChemi, DNR, Israel).

### Dual luciferase reporter gene assay

The targeting relationship between lnc-CDHR and miR-3149 was validated by using the PmirGLODual-Luciferase miR target expression vector (Promega). Co-transfection of 293T cells with miR-3149 mimics or miR-Control was performed with pmirGLO-CDHR or pmirGLO-GAS-mut (miR-3149). We then verified the targeting relationship between PTEN and miR-3149. The wild-type reporter construct pmirGLO-PTEN or the mutant reporter construct pmirGLO-PTEN-mut(miR-3149) was co-transfected with miR-3149 mimic or miR-Control in 293T cells. After 48 hours of transfection, a dual luciferase reporter system (Promega) was used to measure the luciferase activity of each group of cells. Each experiment was repeated at least three times.

### Statistical analysis

Statistical analysis was performed using GraphPad Prism Version 9. One-way ANOVA with Turky’s multi-comparisons test (p≤0.05) was employed to determine statistically significant differences. The measurement data were expressed as mean ± SEM.

### Availability of data and material

The data used to support the findings of this study are available from the corresponding author upon request.

### Consent for publication

All authors read and approved the final manuscript.

## RESULTS

### Identification of hUMSCs

**Figure 1:**
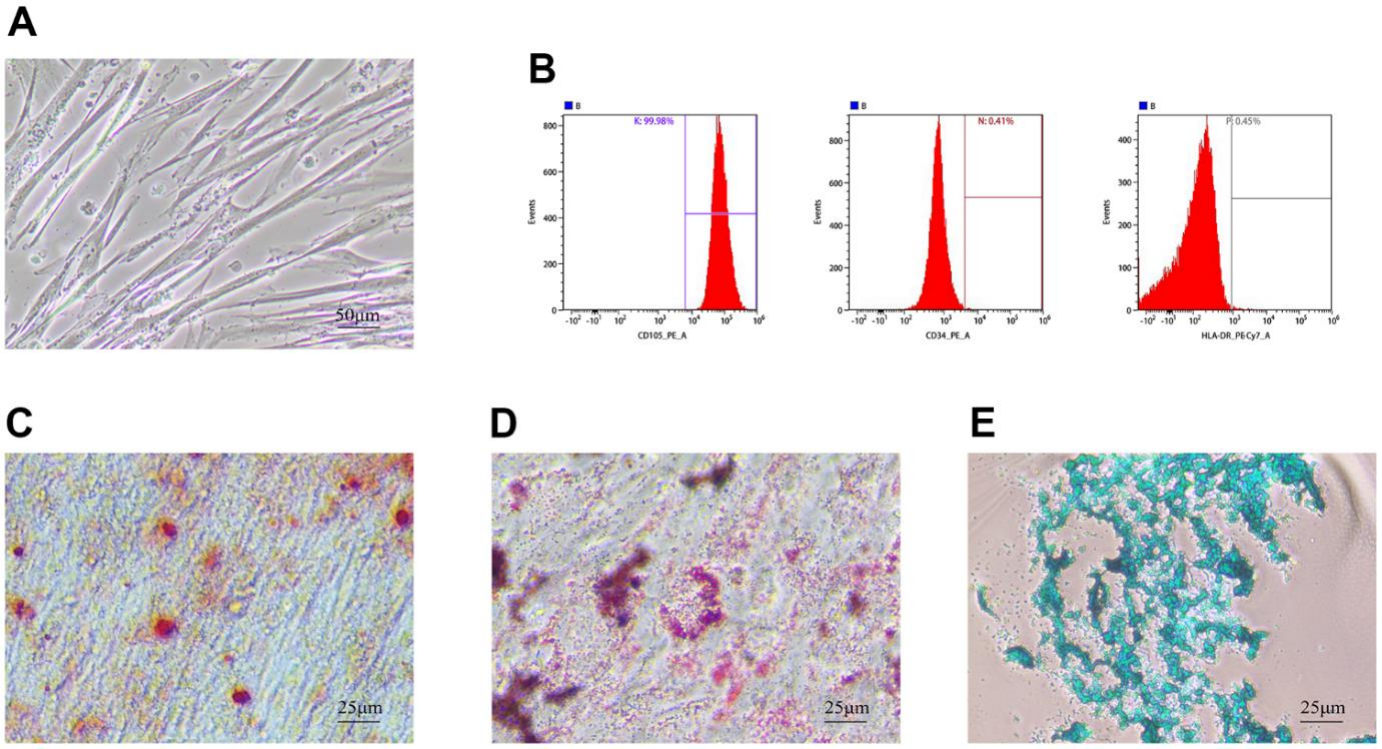
**Identification of hUMSCs.** (**A**) hUMSCs of the 4th-5th generation were observed under the light microscope. (**B**) hUMSC specific surface markers, CD105, CD34, and HLA-DR, were measured by flow analysis. (**C**–**E**) Osteogenesis (**C**) adipogenesis (**D**) and chondrogenesis (**E**) were identified after staining.

Under light microscopy, hUMSCs of the 4th-5th generation grew with a fibroblast-like morphology (Figure 1A). Flow analysis results showed that hUMSCs were positive for surface markers CD105 (99.98%), CD34 (00.41%), and HLA-DR (00.45%) (Figure 1B). After 21 days of induction of osteogenic differentiation, brownish-red opaque calcium nodules were visible by Alizarin Red staining (Figure 1C). After induction of adipogenic differentiation, Oil Red staining showed purple-red lipid droplets distributed in the cytoplasm (Figure 1D). MSC mass induced by chondrogenic differentiation in suspension culture and Alcian Blue staining after frozen section show endoacidic mucopolysaccharides in cartilage tissue (Figure 1E). These results indicated that hUMSCs were successfully identified.

### Identification of hUMSC-Exos

**Figure 2:**
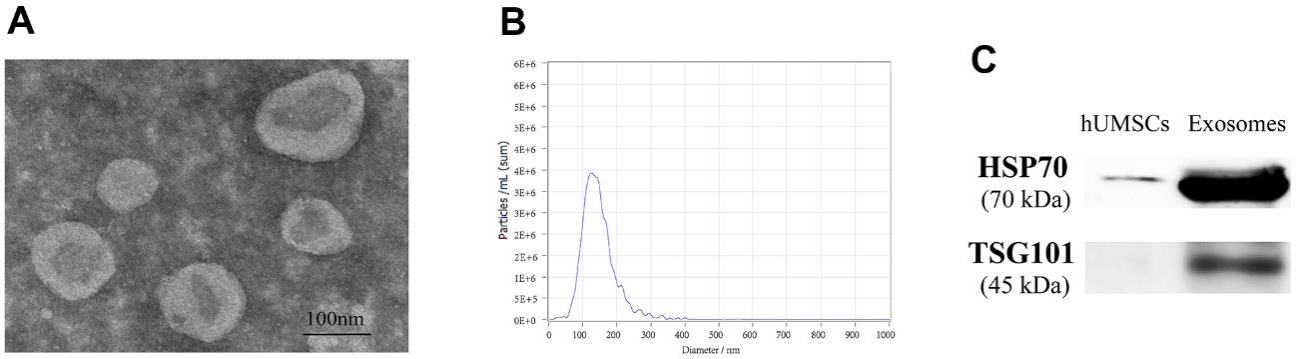
**Identification of hUMSC-Exos.** (**A**) The electron microscopy was used for observing the structure of exosomes. (**B**) The average particle size of exosomes was analyzed through NTA. (**C**) HSP70 and TSG101 proteins were detected by WB.

The concentration of exosomes was 0.3 μg/μl measured by BCA kit after extraction. The exosome electron microscopy showed a clear “teatroid” structure (Figure 2A). NTA analysis showed that the particle number of exosomes reached 6.7×10^10^/ml and the average particle size was 136.5 nm, and the particle size distribution after dilution of 1000 times was shown in Figure 2B. WB results showed that HSP70 and TSG101 were significantly expressed in exosomes compared to hUMSCs (Figure 2C). The above results indicated that hUMSC-Exos was successfully isolated and identified.

### HMrSV5 uptake exosomes derived from hUMSCs

**Figure 3:**
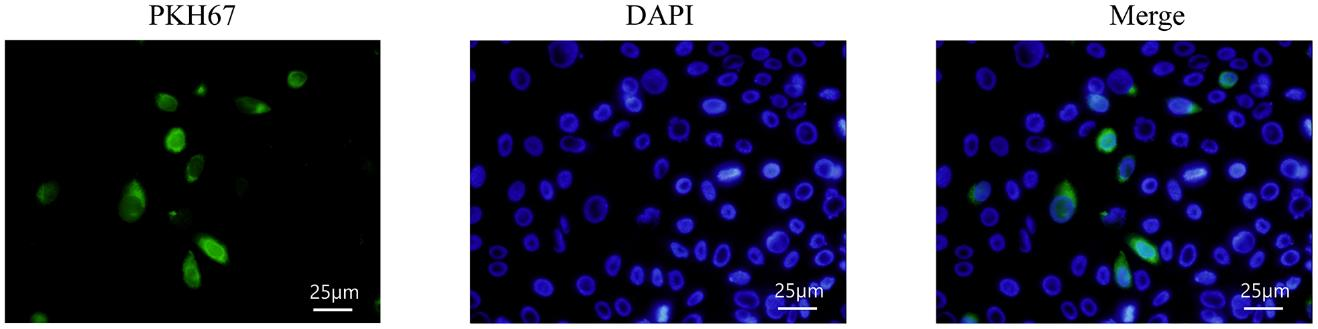
**HMrSV5 uptake hUMSC-Exos.** Exosomes labeled with PKH67 fluorescence were enriched in the cytoplasm of HMrSV5 under an inverted microscope.

To observe whether exosomes mediated the alleviation of HG-induced EMT by hUMSCs in HMrSV5, HMrSV5 was incubated with PKH67-labelled exosomes, and PKH67 fluorescence was seen to be expressed in HMrSV5, indicating that exosomes secreted by hUMSCs could enter the recipient cells (Figure 3).

### hUMSC-CM improves HG-induced peritoneal EMT via exosomes

Peritoneal dialysis involves the osmotic pressure generated by intraperitoneal infusion of hypertonic dialysate, usually in the form of 1.5%, 2.5%, or 4.25% glucose [24], and peritoneal glucose exposure is a key factor in all mechanisms of long-term PD-acquired ultrafiltration failure [25]. According to a previous experiment [26], 2.5% HG applied to HMrSV5 for 48h can successfully induce EMT. After HG stimulation, E-cadherin protein and mRNA expression decreased, while Vimentin and α-SMA increased which were analyzed by internal reference semi-quantitative, demonstrating the success of the EMT model. In our current study, β-actin was selected as an internal reference in WB experiments and GAPDH was used as an internal reference in PCR [27]. Numerous research have shown that exosomes isolated from MSC-CM can mitigate fibrosis progression [7].

**Figure 4:**
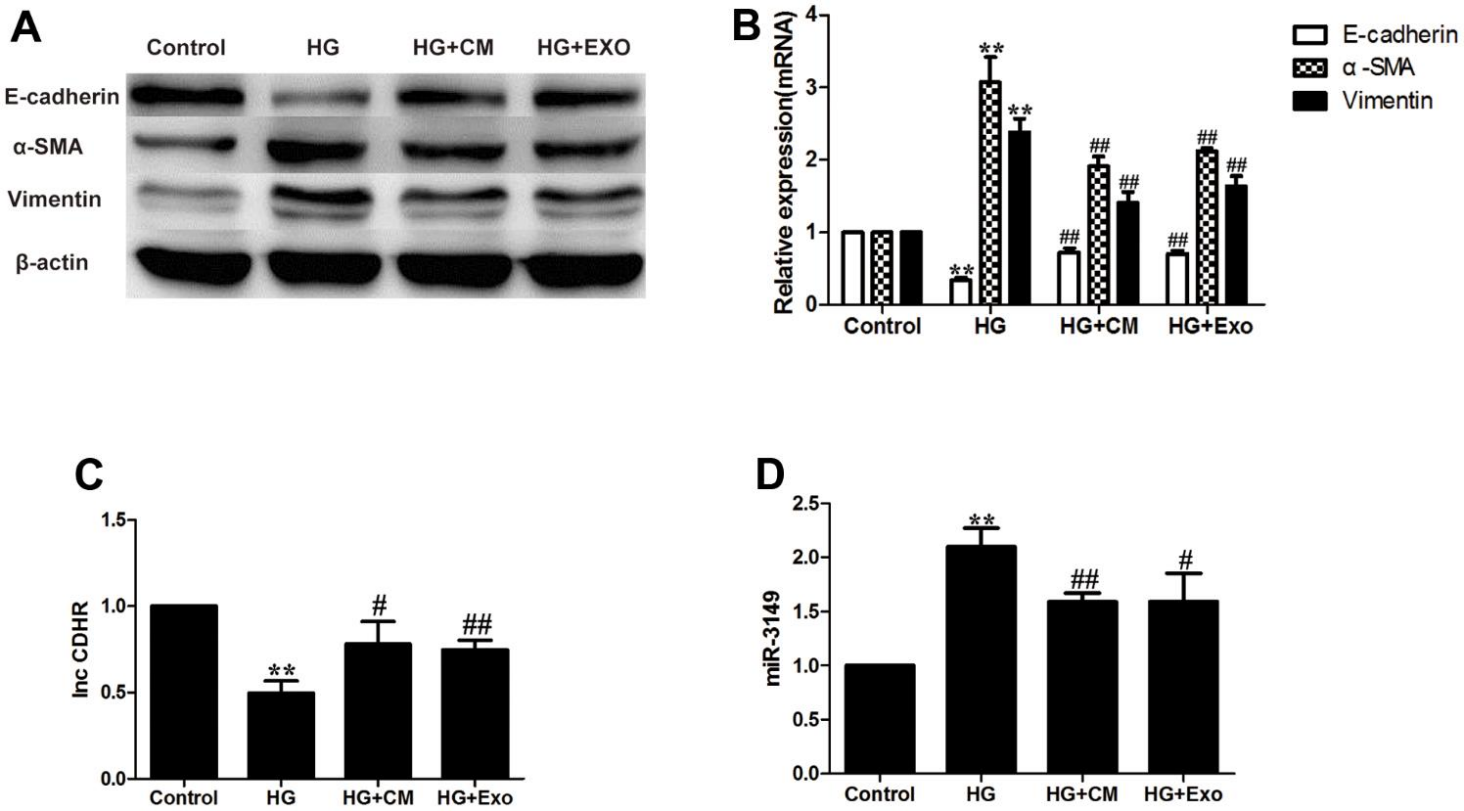
**hUMSC-CM improves HG-induced peritoneal EMT via exosomes.** (**A**, **B**) Expression of EMT markers (α-SMA, Vimentin, and E-cadherin) in HMrSV5 was detected by WB and PCR after hUMSC-CM and hUMSC-Exos treated HMrSV5 which was stimulated by HG. (**C**, **D**) The expression of miR-3149 and lnc-CDHR was detected by PCR. Each value represents the mean ± SEM (n=3) (^**^P<0.01 vs. Control, ^##^P<0.01 vs. HG, ^#^P<0.05 vs. HG).

According to the results, addition of 7.5% hUMSC-CM to the HG group alleviated HMrSV5 EMT. EMT in HG + hUMSC-Exos group was also alleviated, which proved that hUMSC-CM improves HG-induced peritoneal EMT via exosomes (Figure 4A, 4B). Based on our previous exploration, we found that miR-3149 and in-CDHR were also affected by the progression of EMT. HUMSC-CM and hUMSC-Exos can reverse the down-regulation of lnc-CDHR and up-regulation of miR-3149 in the EMT model (Figure 4C, 4D).

### Exosomal lnc-CDHR of hUMSCs alleviates HG-induced EMT by down-regulating miR-3149 and up-regulating PTEN

**Figure 5:**
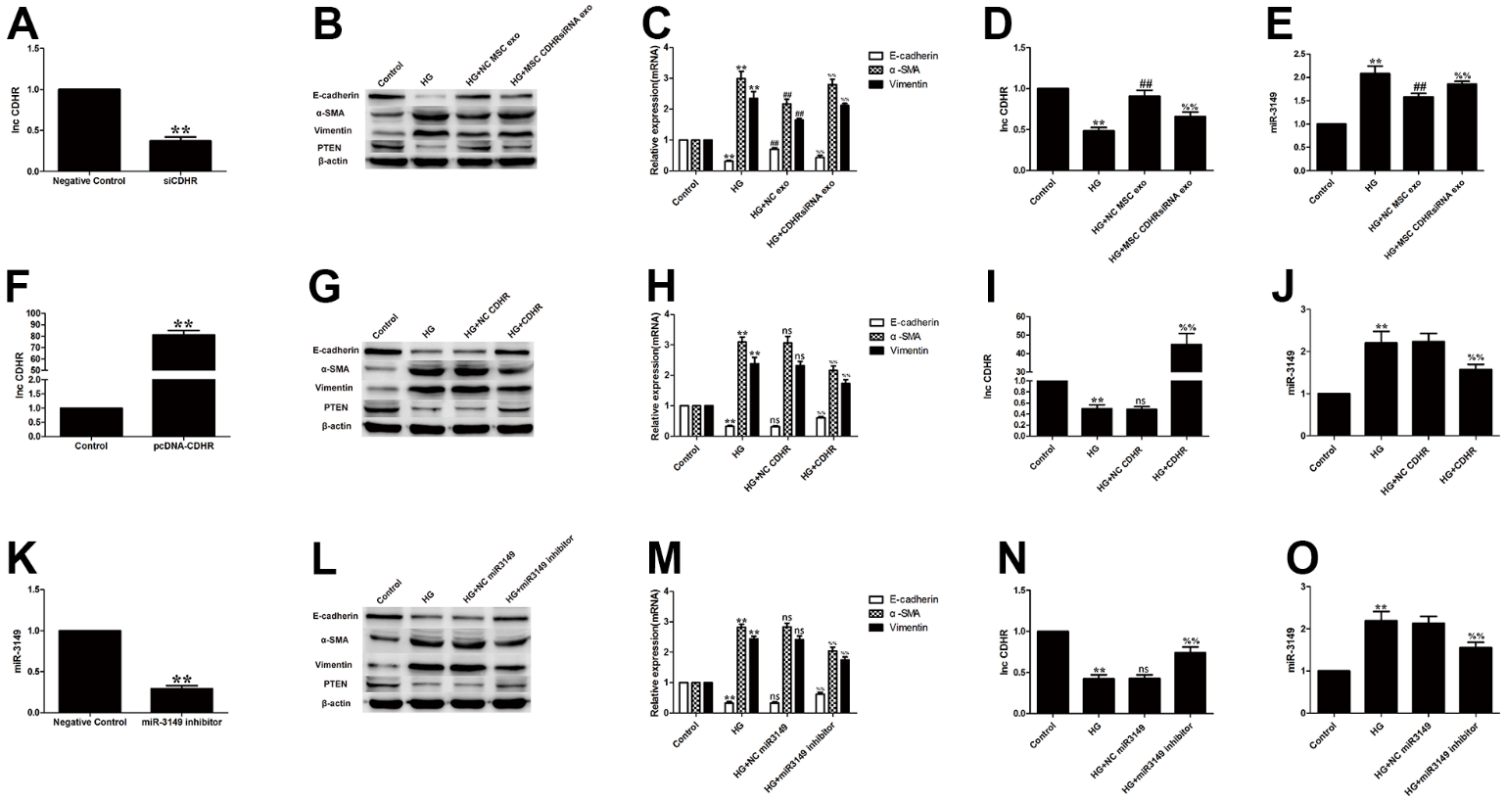
**Exosomal lnc-CDHR of hUMSCs alleviates HG-induced EMT by down-regulating miR-3149 and up-regulating PTEN.** (**A**) Transfection efficiency of exosomal lnc-CDHR extracted from transfected stem cells was detected by PCR. (**B**, **C**) The effect of MSC CDHR siRNA exosomes on the expression of EMT and PTEN in HG-induced HMrSV5 was detected by WB and PCR. (**D**, **E**) Lnc-CDHR and miR-3149 expression were detected by PCR after treatment of HG-induced HMrSV5 with MSC CDHR siRNA exosomes. (**F**) Transfection efficiency of pc-DNA CDHR was detected by PCR. (**G**–**J**) Expressions of Vimentin, α-SMA, E-cadherin, PTEN, CDHR, and miR-3149 were detected by WB and PCR after transfection of pc-DNA CDHR in HG-induced HMrSV5. (**K**) Transfection efficiency of miR-3149 inhibitor was detected by PCR. (**L**–**O**) To illustrate the effect of miR-3149 on EMT, changes of EMT, PTEN, CDHR, and miR-3149 were detected by WB and PCR after miR-3149 inhibition. Each value represents the mean ± SEM (n=3) (^**^P<0.01 vs. Control, ^##^P<0.01 vs. HG, ^ns^ no significance vs. HG, (**C**–**E**) ^%%^P<0.01 vs. HG+NC exo, (**H**–**J**) ^%%^P<0.01 vs. HG+NC CDHR, (**M**–**O**) ^%%^P<0.01 vs. HG+NC miR3149).

To prove that hUMSC-Exos eased EMT by delivering lnc-CDHR to HMrSV5, hUMSCs were transfected with lnc-CDHR siRNA. Exosomes extracted from transfected stem cells were shown to be successfully transfected (Figure 5A). Compared to the HG group, NC MSC exosomes improved the progression of EMT, and PTEN expression was elevated. However, the beneficial effects of hUMSC-Exos were all blocked after treatment of HG-induced HMrSV5 with MSC CDHR siRNA exosomes (Figure 5B, 5C). Furthermore, PCR detection of lnc-CDHR and miR-3149 expression in each group evidenced that low expression of CDHR in exosomes caused high expression of miR-3149 in HMrSV5, which means that the inhibitory effect of lnc-CDHR on miR-3149 was removed (Figure 5D, 5E).

After transfection of pc-DNA CDHR in HG-induced HMrSV5 (Figure 5F), expressions of E-cadherin, PTEN, and CDHR rose, while Vimentin, α-SMA and miR-3149 declined (Figure 5G–5J), further demonstrating that lnc-CDHR can regulate EMT via miR-3149 and PTEN. To illustrate the effect of miR-3149 on EMT, miR-3149 inhibitor was transfected into HMrSV5 (Figure 5K). miR-3149 inhibition significantly reduced EMT and elevated the expression of CDHR and PTEN (Figure 5L–5O). In summary, exosomal lnc-CDHR of hUMSCs alleviates HG-induced EMT by down-regulating miR-3149 and up-regulating PTEN.

### Exosomal lnc-CDHR of hUMSCs competitively bound to miR-3149 and regulated the suppression of target PTEN genes to attenuate EMT in HMrSV5

**Figure 6:**
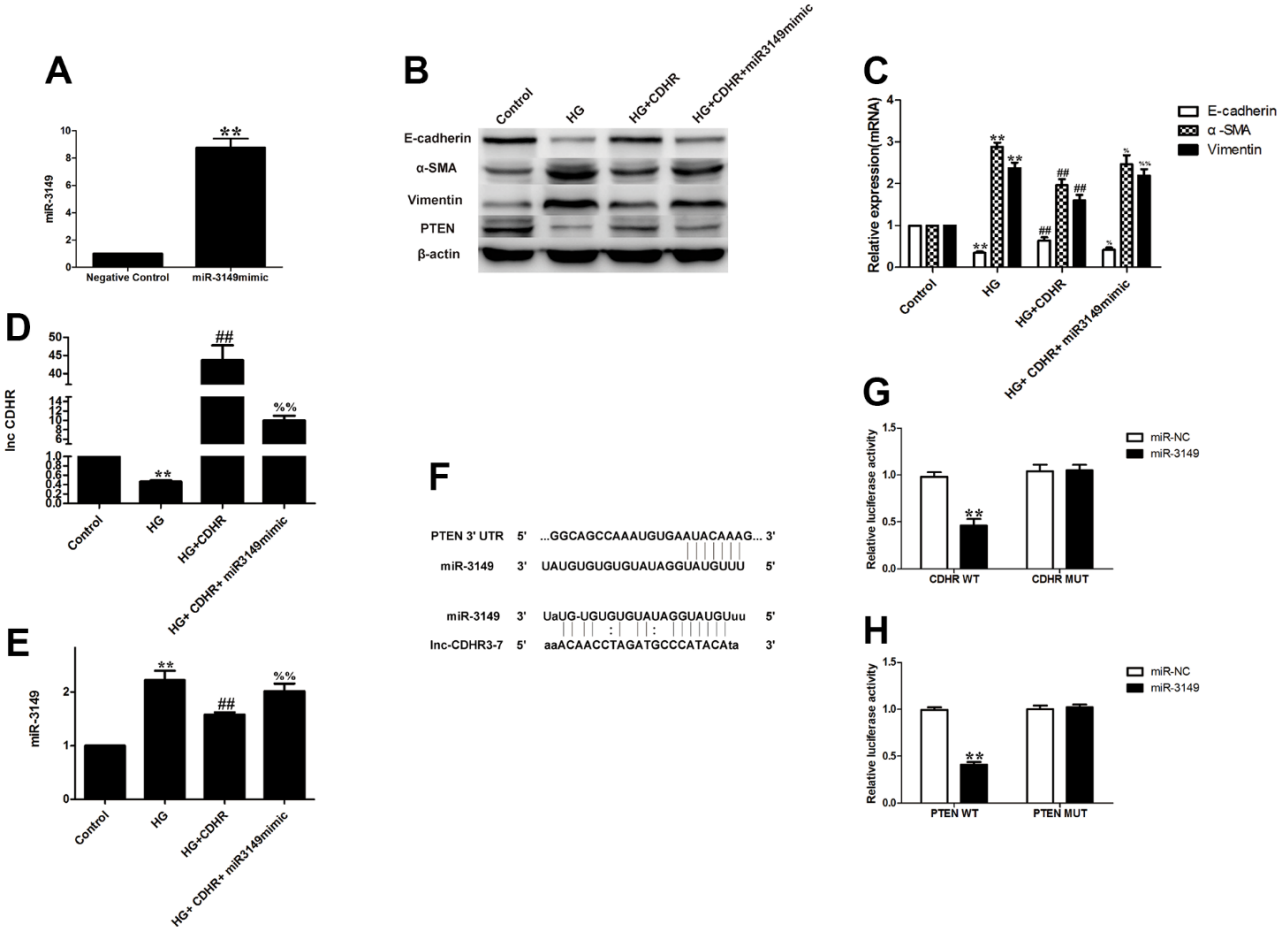
**Exosomal lnc-CDHR of hUMSCs competitively bound to miR-3149 and regulated the suppression of target PTEN genes to attenuate EMT in HMrSV5.** (**A**) Transfection efficiency of miR-3149 mimic was detected by PCR. (**B**–**E**) A rescue experiment was implemented to figure out the relationship between the upstream and downstream of lnc-CDHR and miR-3149. (**F**) Bioinformatics analysis result showed that miR-3149 had a binding site with lnc-CDHR and PTEN. (**G**) Dual-luciferase reporter gene assay was used to analyze the relationship between lnc-CDHR and miR-3149. (**H**) Dual-luciferase reporter gene assay was used to analyze the relationship between miR-3149 and PTEN. Each value represents the mean ± SEM (n=3) (**C**–**E**) ^**^P<0.01 vs. Control, (**G**) ^**^P<0.01 vs. CDHR WT, (**H**) ^**^P<0.01 vs. PTEN WT, ^##^P<0.01 vs. HG, ^%%^P<0.01 vs. HG+CDHR, ^%^P<0.05 vs. HG+CDHR.

To figure out the upstream and downstream relationship between lnc-CDHR and miR-3149, we implemented a rescue experiment. Simultaneous transfection of lnc-CDHR (Figure 5F) and miR-3149 mimic (Figure 6A) into HG-induced HMrSV5 resulted in lnc-CDHR decreasing Vimentin and α-SMA and increasing E-cadherin expression, while elevating PTEN expression (Figure 6B, 6C). In addition, lnc-CDHR inhibited the expression of miR-3149 (Figure 6D, 6E). However, the mitigating effects of lnc-CDHR on EMT were all reversed by miR-3149, demonstrating that lnc-CDHR is located upstream of miR-3149.

Based on bioinformatic analysis, the bioinformatics software predicts that the binding site for miR-3149 is contained in lnc-CDHR and PTEN (Figure 6F). Dual luciferase reporter gene analysis was performed to validate the relationship between lnc-CDHR and miR-3149. The relative luciferin activity was dramatically depressed in the CDHR-WT and miR-3149 mock cotransfected group, and luciferase activity was not inhibited in the CDHR-MUT and miR-3149 mock cotransfected group (Figure 6G). The interaction between PTEN and miR-3149 was then visualized, and the relative luciferase activity was significantly decreased in the PTEN-WT and miR-3149 mock cotransfected group, and the luciferase activity was not restrained in the PTEN-MUT and miR-3149 mock cotransfected group (Figure 6H).

### HUMSC-Exos improve peritoneal EMT through inhibition of the AKT/FOXO signaling pathway

**Figure 7:**
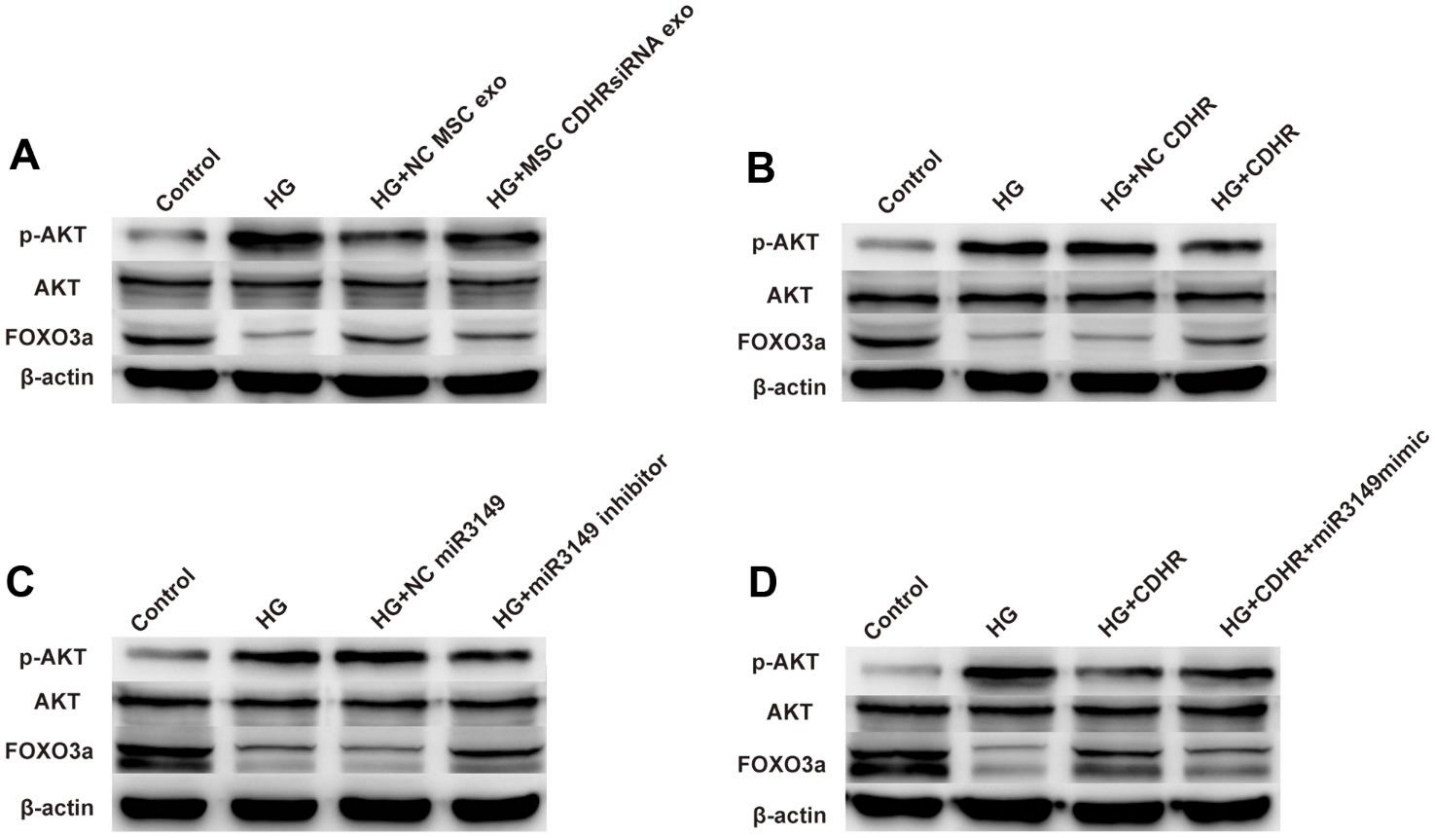
**hUMSC-Exos attenuated peritoneal EMT through regulation of the AKT/FOXO signaling pathway.** (**A**) Exosomes from hUMSCs could suppress AKT/FOXO signaling pathway in HG HPMCs by WB. (**B**–**D**) Exosomal lnc-CDHR from hUMSCs adsorbed miR-3149 to alleviate EMT through the AKT/FOXO signaling pathway.

Compared with the control group, HG treatment increased the ratio of pAKT/AKT and decreased the expression of FOXO3a, indicating that HG induced EMT through AKT/FOXO signaling pathway. Compared to the HG group, exosomes alleviated the decrease in FOXO3a protein levels, suggesting that hUMSC-Exos alleviated EMT through the AKT/FOXO signaling pathway. However, this alleviating effect was impeded by the reduced lnc-CDHR in the CDHR siRNA group, indicating that exosomal lnc-CDHR can relieve EMT via the AKT/FOXO signaling pathway (Figure 7A). Overexpression of lnc-CDHR in HMrSV5 also achieved mitigation of EMT, further validating that AKT/FOXO signaling pathway mediated the remission of EMT by lnc-CDHR (Figure 7B). To explore the relationship between miR-3149 and AKT/FOXO signaling pathway, transfection of miR-3149 inhibitor to HMrSV5 resulted in a distinct reduction in pAKT/AKT and an elevation in protein FOXO3a expression, indicating that AKT/FOXO signaling pathway also mediated the alleviating effect of miR-3149 on EMT (Figure 7C). Finally, to confirm the relationship between lnc-CDHR and miR-3149 on the AKT/FOXO signaling pathway, transfection of both lnc-CDHR and miR-3149 mimic to HMrSV5 resulted in lnc-CDHR alleviating the activation of the AKT/FOXO pathway, and this relief was reversed by miR-3149, demonstrating that lnc-CDHR adsorbed miR-3149 to alleviate EMT through the AKT/FOXO signaling pathway (Figure 7D). These results imply that hUMSC-Exos attenuated peritoneal EMT through regulation of the AKT/FOXO signaling pathway.

## DISCUSSION

Peritoneal dialysis is an important treatment for end-stage renal disease. The risk of infection may be lower in patients with CKD who require urgent initiation of dialysis by adopting PD [28]. PD has lower costs and more patient freedom compared to hemodialysis [29]. Peritoneal dialysis solution mainly uses the high osmotic pressure of glucose to achieve ultrafiltration. On the other hand, HG causes pseudo-hypoxia and hypoxia stimulates myofibroblasts to produce GLUT-1, the latter leading to a further enhancement of intracellular glucose uptake, resulting in a decrease in the ultrafiltration osmotic gradient. A vicious cycle is formed between dialysate glucose exposure, peritoneal fibrosis, and ultrafiltration failure [25]. In addition, TLR2 and TLR4 [30], and the immunomodulatory molecule CD69 [31], have been shown to regulate PF through inflammation. Si M et al. concluded that excessive glycolysis caused EMT and peritoneal fibrosis by analyzing single-cell sequencing data from PD patients [32]. It is evident that prolonged peritoneal dialysis leads to EMT and peritoneal fibrosis as a complex process.

MSCs are now widely investigated in various diseases, such as kidney disease, osteoarthritis, and even COVID-19 [5, 33, 34]. In recent years, researchers have started to focus on the role of MSCs on PF. Fan YP et al. transplanted hUMSCs directly intraperitoneally into rats to effectively prevent peritoneal dialysis/ methylglyoxal-induced PF [35]. Subsequently, MSCs were shown to alleviate PF by modulating inflammation [9, 10]. Some preconditioned MSCs seem to provide better relief of PF. Serum medium culture and SIRT1-modified MSCs enhanced the antifibrotic effect of MSCs by suppressing inflammation [11, 36]. There are only a few relevant studies on MSCs and their supernatants alleviating PF as mentioned above. In recent years, most studies related to the efficacy of MSCs have been attributed to exosomes. In contrast, there are very few studies addressing the protective effects of MSC-Exos on peritoneal EMT and fibrosis so far. We innovatively propose that MSCs alleviates peritoneal EMT via exosomes and explore in depth the possible mechanism of the role of exosomes.

Exosomes as carriers can encapsulate various information molecules, including lipids, proteins, RNA, etc. [5]. 232 lncRNAs were detected to be differentially expressed in the PF group, which indicated that lncRNAs are also involved in regulating the progression of PF [37]. LncRNAs can act as ceRNA sponge miRNAs, thereby regulating the expression of downstream genes [38]. PTEN always is regulated by miRNAs to involve in tissue fibrosis and EMT. Our previous studies reported that miR-21 played a vital role in HG-induced EMT by targeting PTEN in peritoneal mesothelial cells [15, 39]. Also, exosomal miR-21 can target PTEN to cause renal fibrosis in obstructed kidneys [40]. In this research, lnc-CDHR was proved to sponge miR-3149 to regulate and control the expression of PTEN, thus improving the peritoneal EMT. AKT/FOXO pathway is associated with fibrotic processes in some organs, especially the liver [41]. Our study further confirmed that HUMSC-Exos can alleviate peritoneal EMT through the AKT/FOXO signaling pathway. In the present study, hUMSC-Exos was shown to reverse EMT in peritoneal mesothelial cells. Numerous studies have also affirmed the feasibility, efficacy and safety of MSCs and MSC-Exos. However, the optimal source and specific dosing regimen of MSCs and MSC-Exos are inconclusive. The effective concentration of exosomes *in vivo* and *in vitro* experiments still needs to be supplemented and validated. In the future, we will validate the theory in animal models.

## CONCLUSIONS

In our work, hUMSC-Exos regulated HG-induced peritoneal EMT by delivering lnc-CDHR. In particular, lnc-CDHR can adsorb miR-3149 and thus hinder PTEN degradation. Our study also confirmed that hUMSC- Exos can alleviate EMT through AKT/FOXO signaling pathway. This implied that exosomal lnc-CDHR derived from hUMSCs sponging miR-3149 to target PTEN reduced peritoneal EMT through AKT/FOXO pathway. Our study suggests that early intervention of HUMSC-Exos can reverse EMT in HMrSV5, thereby alleviating peritoneal fibrosis and providing a theoretical basis for improving the quality of survival in patients with end-stage renal disease.
